# From Isolation of Phosphate Solubilizing Microbes to Their Formulation and Use as Biofertilizers: Status and Needs

**DOI:** 10.3389/fbioe.2019.00425

**Published:** 2020-01-09

**Authors:** Abdoulaye Soumare, Kenza Boubekri, Karim Lyamlouli, Mohamed Hafidi, Yedir Ouhdouch, Lamfeddal Kouisni

**Affiliations:** ^1^AgroBioSciences Program, Mohammed VI Polytechnic University (UM6P), Benguerir, Morocco; ^2^Université Cheikh Anta Diop, Laboratoire Commun de Microbiologie IRD/ISRA/UCAD, Centre de Recherche de Bel Air, Dakar, Senegal; ^3^Faculté des Sciences Semlalia, Université Cadi Ayyad, Laboratoire of Microbial Biotechnologies, Agrosciences and Environment, Marrakesh, Morocco

**Keywords:** *Actinobacteria* biotechnology, bioformulation, phosphorous, rock phosphate, quality standards

## Abstract

The production of biofertilizers at industrial level is a bottleneck because bacterial strains are generally developed and managed by research laboratories and not by production units. A seamless transition from laboratory to field application is, therefore necessary. This review provides an overview of the constraints that limiting the application or the implementation of *Actinobacteria* based biofertilizers especially in agricultural field and suggests solutions to overcome some of these limits. General processes of making and controlling the quality of the inoculum are briefly described. In addition, the paper underlines the opportunity of biofertilizers alone or in combination with chemical fertilizers. This review also, highlights the latest studies (until June 2019) and focuses on P-solubilization microorganisms mainly *Actinobacteria*. The biotechnology of these bacteria is a glimmer of hope for rock phosphate (RP) bioformulation. Since direct application of RP fertilizer is not always agronomically effective due to its sparse solubility.

## Introduction

The world population will reach at least 9.8 billion by 2050 according to the United Nation Food and Agriculture Organization (FAO) projections (Harold and Reetz, 2016). In order to ensure global food security, at least doubling our current agricultural production is required (FAO, 2017, 2018). To achieve this goal, it is necessary to have very fertile soils or to supplement nutrients in low fertility soils by applying a high amount of fertilizers (Keane, 2009). Until now, chemical fertilizers help in feeding the world by providing three major plant nutrients, nitrogen, phosphorus and potassium (NPK). Approximately 52.3 billion tons of P-based fertilizers are applied annually to maintain available P levels in soil–plant systems (FAO, 2017). Whereas, only, about 0.2%, i.e., <10 μM of this huge amount, is used by plants (Alori et al., 2017; Islam et al., 2019) and the rest is precipitated by metal cations in soil such as Fe, Al, Mg, Ca, etc. The extensive use of P may leads to inevitable depletion of world reserves of rock phosphate (Leghari et al., 2016). This depletion will be concomitant with the increase in the cost of these commercial fertilizer products. Recently, scientists from all over the world have focused their attention on sustainable agriculture by exploiting beneficial microbes in order to increase the contribution of biofertilizers to food and fiber production (Khan et al., 2007; Granada et al., 2018; Yadav and Sarkar, 2019). Biofertilizers are commonly defined as “Preparation containing live microbes which helps in enhancing the soil fertility either by fixing atmospheric nitrogen, solubilizing phosphorus or decomposing organic wastes or by augmenting plant growth by producing growth hormones with their biological activities” (Chaparro et al., 2014; Okur, 2018). Many strains, some of which have interesting biotechnological potential have been isolated from the extensive research programs on plant-beneficial microorganisms (Sivasakthivelan and Saranraj, 2013; Umesha et al., 2018; Nafis et al., 2019). Nitrogen fixers, potassium and phosphorus solubilizers, or their combinations with molds fungi are the organisms commonly used as components of biofertilizers (Mohammadi and Sohrabi, 2012; Pathak and Kumar, 2016). Most of these microorganisms used as biological fertilizers are isolated from the plant rhizosphere where they establish close contact with the roots (Xiang et al., 2012). Rhizobia, Arbuscular mycorrhizal fungi (AMF) and *Frankia* are symbiotic with roots whereas rhizobacteria live in the rhizosphere soil, and/or on the root surface or even in superficial intercellular spaces (Vasconcellos et al., 2010; Stamenković et al., 2018). Nowadays, few microorganisms have been marketed as biofertilizers, and most are in North America, Europe and Asia (particularly India) (Mishra et al., 2017). The low R&D funding in this area, poor quality of such products and low level of adaptability to the farmer's needs could explain their low usage despite their reputation of being easy to use, non-toxic, cheap, eco-friendly and sustainable (FAO, 1991; Date, 2001; Hungria et al., 2005; Shravani et al., 2019). This has resulted in a lack of credibility in these products and makes their sale difficult (Stamenković et al., 2018). Therefore, scientists and biofertilizers manufacturers need to collaborate in order to overcome the bottlenecks in the process of bacterial bioformulation. In this regard, *Actinobacteria* constitute promising source of novel biofertilizers in sustainable agriculture. Indeed, *Actinobacteria* have the advantage of surviving in very competitive environments and produce spores during unfavorable conditions (Nandimath et al., 2017). These filamentous organisms can bridge water free gaps between soil particles to move to a new nutritional site (Olanrewaju and Babalola, 2019). Their morphology also gives them a much higher surface to volume ratio and improves their ability to solubilize phosphorus. Therefore, there are immense opportunities for using phosphate and potassic rocks to produce biofertilizers based on *Actinobacteria*. The development of these biofertilizers could optimize the use of natural phosphate in the world. This comprehensive review aims to (i) make a current assessment of the works that have been done in microbial biotechnology research in particular the P-solubilizing *Actinobacteria* and (ii) to identify the major limitations and problems in their bioformulation before proposing future research pathways that can contribute to an effective industrialization.

## Biofertilizers as Complement to Chemical Fertilizers

Microbes are very small but very powerful and useful. In fact, all the elements brought as chemical fertilizer can be provided by microorganisms, especially Nitrogen (N), Phosphorous (P), and Potassium (K) (Nath et al., 2018). Most of the time, these elements are sufficiently available in the soil (NPK) or in the atmosphere (N) but in an unassimilable form for plants and animals. Rhizobia, Frankia and free-living fixer convert dinitrogen gas (N_2_) into usable form (NH4+ mainly) for plants uptake, while Arbuscular Mycorrhizal fungi, potassium and phosphate solubilizing microbes dissolve mineral and/or organic sources to make P and K available to plants (Rashida et al., 2016; Bargaz et al., 2018). AMF can also supply other macro and micronutrients such as Fe, Mg, K, Ca, Cu Mn, Zn etc. (Chen et al., 2017). The other important benefits of microbes are their ubiquitous distribution and adaptation to environments (Qu et al., 2019). Some microbes are extremophilic and extremotolerant to various environmental factors such as pressure, drought, salinity, and pH (Rampelotto, 2010; Banga et al., 2018). In the current context where sustainable farming systems are much needed, the use of these plant-friendly microorganisms as biofertilizers is more and more urgent (Yadav and Sarkar, 2019). In fact, the high cost of chemical fertilizers formulation and the difficulties of meeting their demand have encouraged scientists to develop biofertilizers as a solution to replace or at least partially substitute chemo-synthetic and chemical fertilizers (Vaxevanidou et al., 2015). However, the task is not easy because chemical formulation in agro-products is standardized for long-term storage, resistance to abuse of application and ease of use by farmers (Bashan et al., 2014). Thus, biofertilizer need to meet these standards, and produce evident effects like those produced by chemical fertilizer, otherwise it will be difficult to convince farmers, with very low incomes to use them. This means that, in the absence of efficient biofertilizers with consistent quality, their contribution, compared to the chemical fertilizers will remain too low. However, biofertilizers have the ability to slowly release nutrients from a source according to the crop's need and complement other minerals and growth factors an advantage over chemical fertilizers (García-Fraile et al., 2015; Yadav and Sarkar, 2019). Some researchers have tried to test mixed fertilizers combining biofertilizers with organic and/or chemical fertilizers (Cisse et al., 2019). Chen (2006) showed that a cocktail of beneficial microorganisms in compost and urea could help to save the input of chemical or organic fertilizers and decrease P accumulation in the soil. Similar results were reported by Mondal et al. (2015), after combining chemical fertilizers with biofertilizers and vermicompost. They concluded that vermicompost can substitute at least 25% of NPK fertilizer amount and help to increase farmers' incomes. The authors Latha and Jeyaraman (2014) found that the use of chemical fertilizers alone in brinjal (*Solanum melongena* Linn.) crop had a higher cost-benefit ratio compared to the combination of biofertilizers and chemical fertilizers. Other positive effect of phosphorus biofertilizer in combination with chemical fertilizer on wheat (Farsani et al., 2013), rice (Naher et al., 2016), and soybean (Munda et al., 2015) have been published. Farmers' can therefore get more profit by combining both fertilizers. In view of these results, the urgency to formulate high efficient fertilizers which combine chemical, organic material, and microbe is considerably high and will become more efficient in the future.

Currently, the biofertilizers market is valued around $1.57 billion and is expected to reach $ 1.88 billion by 2020, while the global fertilizer market need will be around $245 billion. Biofertilizers based on P-solubilizing bacteria account for only 14% of this market (Market data forecast, 2018). The main P-solubilizing production units are currently in India and most of them are using strains belonging to *Bacillus* genus (Table 2). Limited information is available on the production capacity and most of these units are of local scale production. Therefore, P-biofertilizers still have a lot to gain in this market. However, there is an overdue need to understand their limiting factors and emphasize the great opportunities of their use.

## Obstacles of Biofertilizers to Overcome

### Relative to the Carrier

The weak point of commercialization is often the performance of the microbial inoculum (Mishra and Arora, 2016). The most common barriers of inoculants commercialization are the formulation inadequacies (Bashan et al., 2016a). Indeed, a micro-organism may be efficient in laboratory and/or greenhouse conditions but formulating that organism into an adequate carrier is a difficult step (Reddy and Saravanan, 2013). Hence, the choice of the carrier material is fundamental because carrier (inoculant) is the sole delivery vehicle of live microorganisms from the production unit to plants in the field. A good carrier should be: (i) nontoxic to organisms (ii) easily pulverized and sterilized (iii) able to carry exceptionally high microbial numbers (iv) easily available and cost effective, and (v) should have a good absorption capacity (Mishra and Arora, 2016). The carrier materials can be of various origins: inorganic, organic, or synthesized from specific molecules. However, whatever its origin, the carrier should keep microorganism's viability during storage in the farmer's warehouse and should have a long shelf life and stability (Bashan et al., 2016a). Research must therefore focus on this step-in order to find carriers that meet these needs. Many materials such as charcoal, peat, lignite, vermiculite, farm coal manure mix, charcoal farm yard manure (FYM) mixture, charcoal-soil mixture and kaolin have been identified as suitable carriers (Bashan, 1986; Berninger et al., 2018). However, these carriers are very prone to contamination after formulations that can reduce the shelf life of the inoculant after autoclaving (Brahmaprakash and Sahu, 2012). The innovations offered by materials science and engineering technologies help to avoid these limitations. For instance, formulations based on alginate beads encapsulation offer advantages of delivering microorganism at the right and precise concentration without losing efficacy (Bashan et al., 2014; Shang et al., 2019) and keeping the quality of inoculant products (Deaker et al., 2011). This technique which lately has become popular in the pharmaceutical, nanotechnology, medicine, aquaculture, and cosmetics industry, ensures the inclusion of liquids, gases or fine solid particles with natural or synthetic polymer (Bashan et al., 2014). The polymeric, deserves attention, because many studies have reported that alginate beads improve cell survival with remarkable flexibility, biodegradation and biocompatibility (Herrmann and Lesueur, 2013; Dragostin et al., 2017). Nevertheless, this approach need to be optimized especially the type of alginate, and the diameter of the beads to obtain the best function after bio-formulation by encapsulation (Bashan et al., 2016a).

Other research focuses on inorganic carrier such as water-in-oil emulsions (Molet-Rodríguez et al., 2018). The principle of this method is to mix water and oil, so, oil is dispersed as droplets in a continuous water phase. The microorganisms are trapped in water droplets and are protected by a physical barrier of emulsifiers. Sometimes, the process can be improved by adding a surfactant i.e., surface-active agent. This technology is widely used in pharmaceutical and cosmetic industries and cancan be adapted to microbial bioformulation (Malusá et al., 2012). However, a certain number of parameters such as oil type, oil density and, emulsion stability must be mastered (Molet-Rodríguez et al., 2018). To overcome these obstacles, collaboration between researchers in the field microbiology and materials science is necessary because bioformulation is at the crossroads of these two disciplines.

### Relative to Quality Standards

Quality standards are the major concern of microbial biotechnology. Two important characteristics define the quality of a bio fertilizer: the presence of the recommended strain in active form and required number. If any of these characteristics is missing in the product, the biofertilizer could be termed as sub-standard (Ghosh et al., 2001). Currently, the quality control framework is not well-defined. Furthermore, the present quality standards as prescribed by regulatory agencies of a country, do not authenticate the strain used for commercial production (Balachandar, 2012). As a result, most of the inoculants produced in the world are of relatively poor in quality (Thuita et al., 2018). To solve this problem, international standards through the ISO Standards should be established and be adapted in the production and use of these biofertilizers. Although efforts to regulate the industries have been made by some countries such as India, Canada, Brazil, China, etc., there are still insufficient (Hungria et al., 2005). In China, the number of cell forming units (CFU) is considered as the main parameter in assessing the quality of the different kinds of biofertilizers (Malusá and Vassilev, 2014). In the European Union, quality parameters for biofertilizers are not yet standardized and they vary from one region to another (Malusá and Vassilev, 2014). Currently, India seem to have the most standardized and functional legal framework related to biofertilizers (Malusá and Vassilev, 2014; Manashi et al., 2017).

### Quality Control Procedure

**Figure 1:**
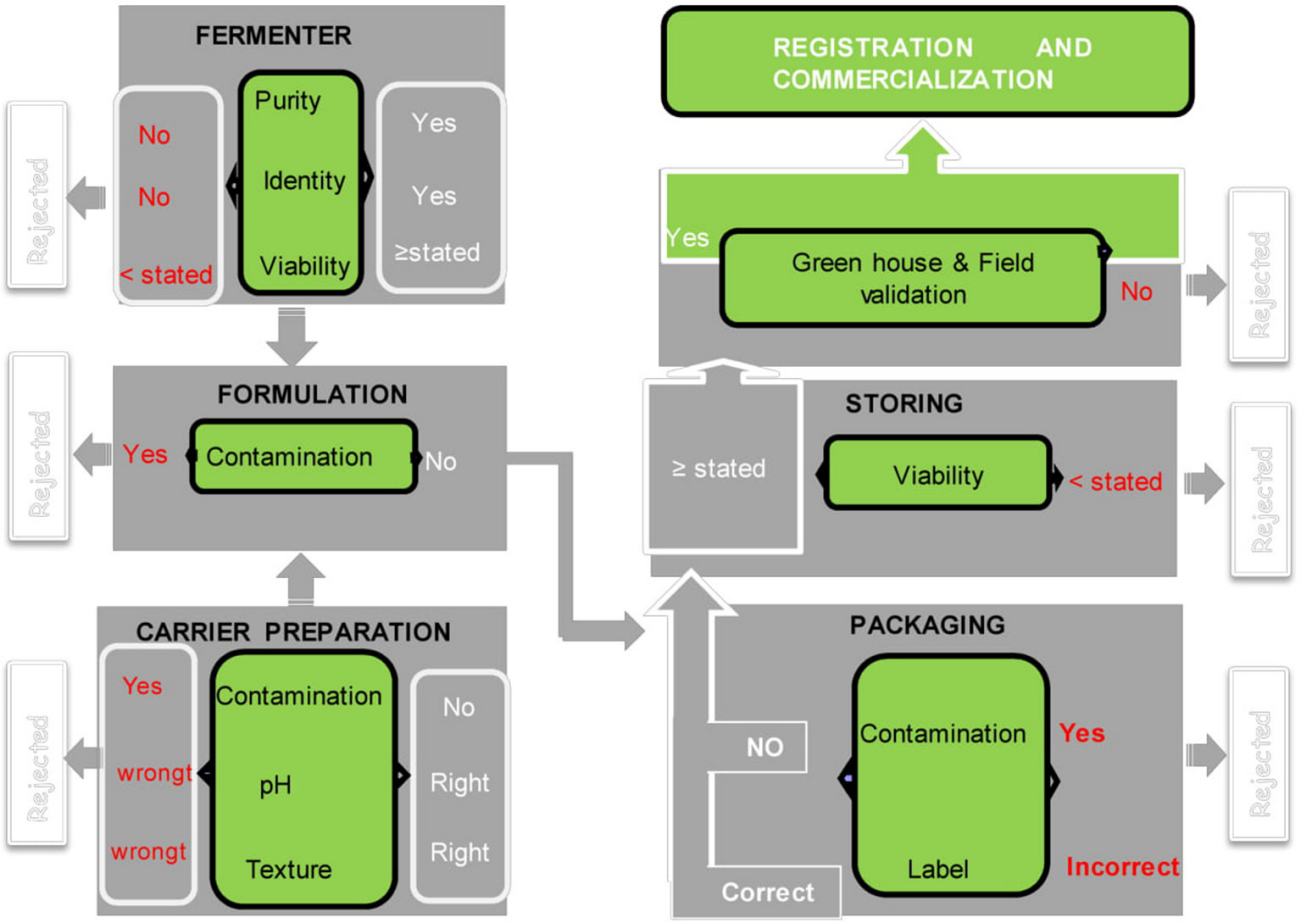
Quality control in the biofertilizer manufacturing process [modified from García-Fraile et al. (2015), Permission acquired from the author]. Writing in red means that the quality control is not compliant, and the project is rejected the writing in white corresponds to the compliant product. The diagram shows that bio-formulation has six main steps before commercialization and four each stage, a quality control is necessary.

To ensure product safety, efficacy, and conformity to prescribed standards, quality control and regulation of bio-fertilizers are important steps (Arora et al., 2016). Inoculum quality control should be done at different stages: screening and efficiency at laboratory and the greenhouse scale by researchers, formulation including choice of support, packaging, and storing at industrial scale. Each step requires expertise, and everything must be governed by standards (under regulation) (Figure 1). Unfortunately, all biofertilizer production units do not follow this procedure due to lack of technical back up.

### Techniques' for Strain Identification

**Figure 2:**
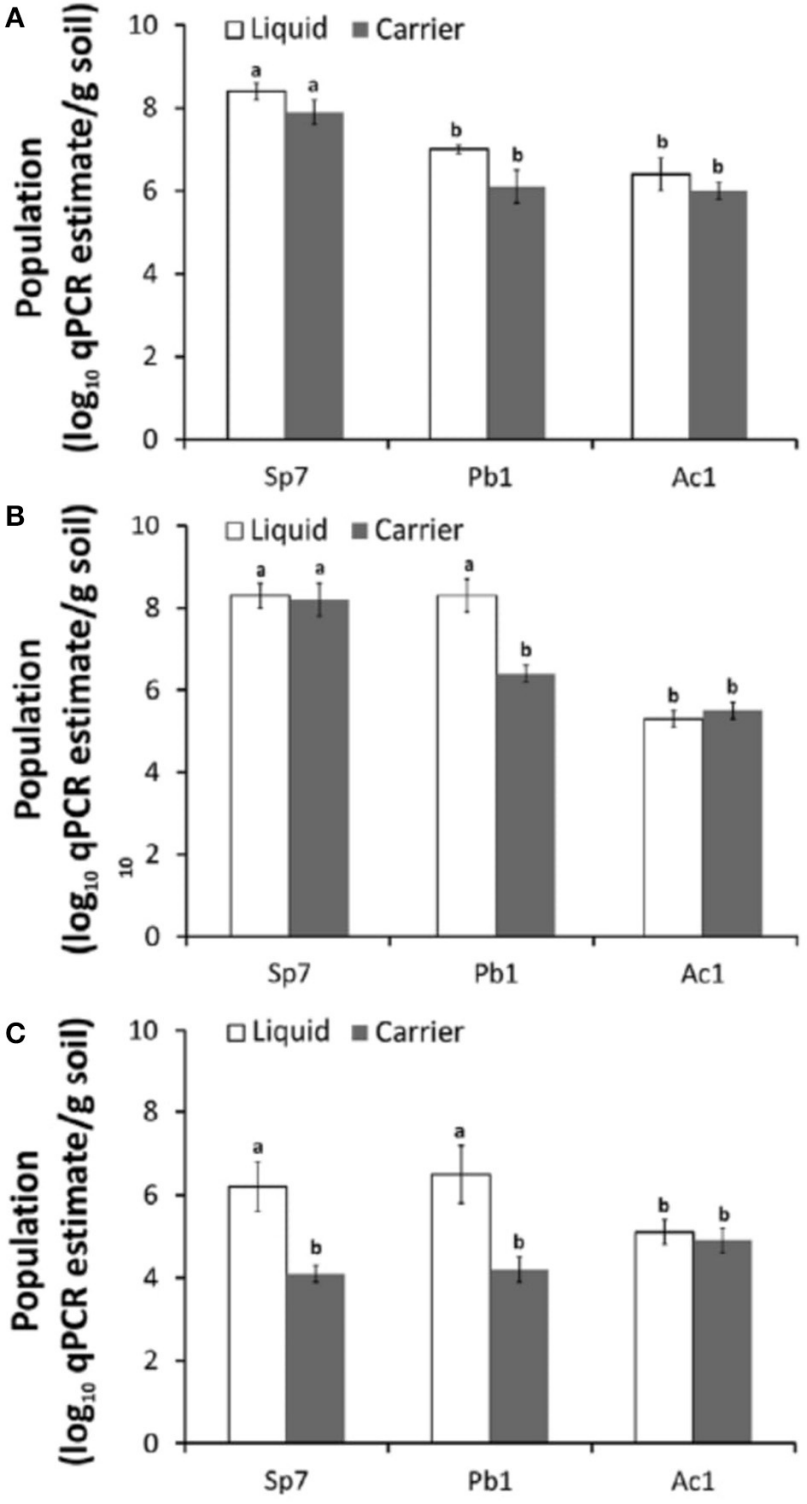
PCR targeting SCAR markers of biofertilizer strains for strain-level authentication and qualification in maize rhizosphere at different growth stages 15th **(A)**, 30th **(B)**, and 60th **(C)** day. Error bar indicates with letters “a” and “b” (ANOVA and DMRT test; *p* < 0.05) (Reddypriya et al., 2019) Reproduced with permission.

Until now, at the international level, there are still no quality control procedures and guidelines for monitoring microbial cells and activity during the production and formulation of inoculants. Many enumeration methods exist, each with its advantages and limitations (Table 1). Usually, the number of living cells is counted by spread plate or drop plate methods or Most Probable Number Method (MPN). However, all these enumeration methods are not specific to a particular strain and are not suitable enough to detect low population levels (Table 1). Additionally, contaminants have some effect on counting protocols and they do not allow identity of target strain. Molecular biology offers techniques for detecting the presence and abundance of specific microorganisms whether in the soil, rhizosphere or in a commercial inoculant, with high sensitivity. Sequence Characterized Amplified Region (SCAR) marker-based fingerprinting produces very good results as demonstrated by a recent research we carried out (Reddypriya et al., 2019) and those of Couillerot et al. (2010) and Reddypriya et al. (2016). Our results showed that, in addition to authentication, quantitative PCR and SCAR marker allow for assessing the cell loads per g/ml of inoculants (Figure 2). In this study, the presence and abundance of *Azotobacter chroococcum* (Ac1), *Bacillus megaterium* (Pb1), and *Azospirillum brasilense* (Sp7) in biofertilizer-inoculated maize rhizosphere soil was detected by small DNA fragments of 299, 375, and 584 bp, respectively. These results clearly showed that multiplex PCR and real-time PCR targeting SCAR markers could be applied in quality control of commercial biofertilizer production, to guarantee quality product for farmers satisfaction. This work can be extended to all potentially usable strains as biofertilizer.

**Table 1:** Main manufacturer of biofertilizer based on P-solubilizing microbes.

**Product unit/ county**	**Name of product**	**Component**	**Form (s)**	**Performance declared by the manufacturer**	**References (Web ID)**
TNAU Agritech Portal/INDIA	Phosphobacteria and Phosphatika	2 bacterial^*^ and 2 fungal species	Powder/Liquid	Increase yield 5–30%	http://agritech.tnau.ac.in
Monarch Bio-Fertilisers and Research Centre/INDIA	Phosphobacteria	Bacteria ^*^	Powder	Dissolve 30–50 kg of phosphorous/hectare	http://www.monarchbio.co.in/bio_fertilizers.html
SAFS Organic Enterprises/INDIA	Phosphobacterium	Bacteria^*^	Powder/ Liquid	No indication	https://www.indiamart.com/safsorganicenterprises/bio-fertilizer.html#bio-fertilizer-phosphobacterium
Agro bio tech Research Centre LTD/INDIA	Phosphobacteria	*Bacillus megaterium var. phosphaticum*	Powder/ Liquid	No indication	http://www.abtecbiofert.com/products.htm
Ajay Bio-Tech (India) Ltd/*India*	Biophos	Bacteria^*^ & Fungi	Powder (spore)	No indication	https://www.linkedin.com/company/ajay-biotech-india-ltd/?originalSubdomain=fr
International Panaacea Limited /India	Phosphofix	Bacteria^*^	Liquid	Reduce 25–30% phosphatic fertilizer requirement	https://www.iplbiologicals.com/
Varsha Bioscience And Technology India Private Limited/INDIA	Phosphomax	*Bacillus megatherium*	Powder	Increase Crop yield by 15–25%	http://www.varshabioscience.com/products/phosphomax.html
T. Stanes & company Limited/India	Symbion-P	*Bacillus megaterium* var. phosphaticum.	Liquid	Saves up to 50% over the cost of phosphorous chemical fertilizer	http://www.tstanes.com/products-symbion-p.html
Novozymes Biologicals Limited/Canada	Jumpstart LCO	-*Penicillium bilaii*	Powder/Granular	Solubilize 8.25 kg/ha	https://www.novozymes.com/en/advance-your-business/agriculture/crop-production/jumpstart
AgriLife/India	P Sol B^®^- BM	*Bacillus megaterium*	Powder (spore)	No indication	http://www.agrilife.in/bioferti_psolb_bm.htm

**Table 2:** Reliability comparison of some methods for detection and enumeration.

**Detection method**	**Sensitivity**	**Advantages**	**Limits**	**References**
McFarland turbidity standard	Low	Non-destructive, rapid and low cost	- Size of microorganisms influence bacterial concentration - Not usable for identification - Not possible to distinguish between live and dead cells	Guo et al. (2017)
Plate count	Low	Enumerate microorganisms	- Time-consuming and laborious - Not usable for identification - Only for culturable cells - Important limitations for anaerobic bacteria	Emerson et al. (2017) Clais et al. (2015)
Most probable number (MPN)	Low	Enumerate microorganisms	- Only for symbiotic microbes - Not usable for identification - poor repeatability	Deaker et al. (2011)
MPN-PCR	Meduim	- Specific detection and enumeration - Applicable to non-culturable microorganisms	- Dependent on specificity of probes	Bonny et al. (2018)
Cell count by microscopy	Meduim	Enumerate microorganisms	- Time-consuming and laborious - Not possible to distinguish between live and dead cells - Expensive equipment and/or consumables - Detection not always possible	Xie et al. (2018)
Colony immunoblotting	Meduim	- Specific detection and enumeration - Non-destructive an inexpensive method	- Only for culturable cells - Identifiable markers required	Kecskés et al. (2009)
Scar marker/Quantitative real-time PCR	High	- Large number of samples can be screened at a time - Good repeatability - Specific and reproductible - Detection and Quantification - Applicable to non-culturable microorganisms	- Expensive and sophisticated, equipment laboratory is required - Requires specific primers designed from a specific gene - Dependent on specificity of probes	Reddypriya et al. (2019)
Next generation gene sequencing systems	High	- Detects the presence of different microorganisms in a sample	- Expensive - Required bioinformatic analysis	Abbasian et al. (2018)

Previously, colony immunoblotting technique was evaluated for the specific detection and enumeration of *Citrobacter freundii* in sterilized or unsterilized carrier media in commercial products (Kecskés et al., 2009). Today, the sensitivity of microbial identification and enumeration in a sample is improved by next generation sequencing Technique (Abbasian et al., 2018). However, the analysis in Table 1 shows that it is currently difficult to find a technique that meets all the desired criteria, i.e., reliable, rapid, inexpensive, and relatively easy to use. Molecular techniques generally have good reliability but require expensive and sophisticated laboratory equipment and technical expertise (Table 1). From this comparative analysis, we conclude that SCAR marker studies have more advantages and deserve to be extended to all microorganisms used in bioformulation.

## Potential Features of Phosphate Solubilizing *Actinobacteria*

Several strains cited in the scientific literature as potentially useful do not appear in the commercial market, perhaps because of inappropriate formulation (Bashan et al., 2014). Unfortunately, they are lost or forgotten. For example, in Africa, lots of efficient isolates belonging to *Azospirillum, Rhizobia, Cyanobacteria, Azotobacter* (for N_2_ fixation), *Pseudomonas, Bacillus, Aspergillus, Penicillium* (for P solubilization and PGPR), *Bacillus* (K solubilization), and arbuscular mycorrhiza (for P mobilization) are described in literature (Hamdali et al., 2012; Ndoye et al., 2013; Barka et al., 2016; Berruti et al., 2016; Hassimi et al., 2017; Soumare et al., 2017) but none of them (or only very few) has been formulated and marketed. This observation can be extended to many other countries and even all over the whole world. There are many reasons that might have led to this situation, but lack of funding is one of the most important part. Grant applications regarding bio-formulations are seldom successful. The lack of private sector involvement in the production and formulation of the inoculum is perhaps the missing link in the chain. The contribution of phosphate producing companies from rock P is a necessity for bio-formulation of phosphate solubilizing microbes (PSMs). This investment will be beneficial for these companies because it will allow them to diversify their offer to farmers. The phosphorus importance for plant growth and role of bacteria to make it available is presented in Box 1.

Box 1 **Importance of P in live and role of bacteria to its availability**

Phosphorus is considered as the second most vital plant nutrient as well as second most deficient after nitrogen (Leghari et al., 2016). It is highly abundant in nature as rock phosphate. The global resources of rock phosphate are of the order of 163,000 million tonnes. Africa contains about 41%, USA has 21%, USSR 13%, the Middle East 10%, Asia 8%, South America 3%, New-Zealand and Oceania together accounts for only 2% and entire Europe <1% (Kumari and Phogat, 2008). Without P, life would not exist because it is a vital component of cell walls, DNA, RNA, and ATP to transport energy to the brain. In crop growth, there is no substitute for phosphorus and phosphorus cannot be synthetically manufactured. It is available for plants in the form of orthophosphate ions H_2_${\text{PO}}_{4}^{-}$ and ${\text{HPO}}_{4}^{2-}$ (Leghari et al., 2016). Plants require this phosphorus for cell growth, the formation of fruits and seeds and ripening (Figure 4). Unfortunately, P is one of the least biologically available nutrients. The fact that certain soil microbes are capable of dissolving relatively insoluble phosphatic compounds has opened the possibility of inducing microbial solubilization of phosphates in the soil. The maximum level of phosphorus solubilization potential of Actinomyctes isolates selected from tropical soils was established around 1,727 μg/ml of Ca-P and 48.0 μg/ml of RP (Leghari et al., 2016).

The use of phosphate solubilizing microbes (PSMs) will efficiently increase fertilizers uptake by mobilizing insoluble phosphorus in the fertilizers and in soils to which they are applied (Suleman et al., 2018). In fact, chemical P fertilizer is subjected to chemical fixation (see Box 1) in soil with some metal cations (Viani et al., 2014) and losses by leaching (Fortune et al., 2005). Combined application of rock phosphate with slow-release PSMs would be a sustainable solution in the modern agriculture. Thus, natural RP is a valuable alternative and less expensive natural source for phosphate fertilizers (Biglari et al., 2016). Sane and Mehta (2015) showed that phosphate solubilizing bacteria and fungi in co-inoculation increase the mobilization of rock phosphate and plant growth. Authors Zaidi et al. (2017) and Ding et al. (2019) reported that PSMs isolated from the rice rhizosphere released 22–826 μg P mL^−1^ in *in vitro* test. Among the PSMs, *Actinobacteria* (Actinomyces) are of special interest since these filamentous sporulating bacteria (see details in Box 2) are able to develop in extremely different soils and offering a unique opportunity for biotechnological application (Mengual et al., 2016).

Box 2 **Features and taxonomy of *Actinobacteria***

*Actinobacteria* are a distinct group of bacteria that are widely distributed in nature. They are gram-positive bacteria with high G+C content. Their taxonomy is extremely complex, they were first divided into two groups namely, Streptomyces and non-Streptomyces or also known as rare A*ctinobacteria*. In volume 5 of the Bergey's, the phylum *Actinobacteria* is divided into 6 classes namely Actinobacteria, Acidimicrobiia, Coriobacteriia, Nitriliruptoria, Rubrobacteria and Thermoleophilia. According to the recent classification of Barka et al. (2016), the class Actinobacteria comprises five subclasses, 10 orders, 56 families, and 286 genera.

Actinobacteria are organisms with characteristics common to both bacteria and fungi but possessing distinctive features to delimit them into a distinct category. They are unicellular like bacteria but produce a mycelium which is non-septate. They resemble fungi because they are adapted to life on solid surfaces and they can produce mycelium and dry spores like most fungi (Kalakoutski and Agre, 1976). On culture media, they have different cultural characteristics (Figure 5) and occur in the soil in the spore stage as well as in the mycelial stage (Figure 5). Their mycelial growth form creates the potential for the formation of large networks (Krsek et al., 2000). They are typically present at densities in the order of 10^6^-10^9^ cells per gram of soil and they represent more than 30% of the total population of soil microbiomes (Polti et al., 2014). *Actinobacteria* play an important role in soil and in plant interaction because of their ability to produce a large number of secondary metabolites, many of which possess antibacterial activity (Lazzarini et al., 2000). Actinobacteria produce approximately two-thirds of the known antibiotics produced by all microorganisms (Dhanasekaran et al., 2012; Feina et al., 2019). This nutrient cycling capacity makes them an ideal candidate for natural fertilizers (Jog et al., 2016; Olanrewaju and Babalola, 2019). In addition, the ubiquitous presence and possible global distribution of many of these actinobacteria suggest a great environmental tolerance. *Actinobacteria* could be used for the formulation of novel biofertilizer and bio-control products constituted by spores and/or mycelium in association with pulverized rock phosphate (Reponen et al., 1998; Nandimath et al., 2017). Unfortunately, this great potential interest of *Actinobacteria* is almost unexplored in Africa and all over the world.

**Figure 3:**
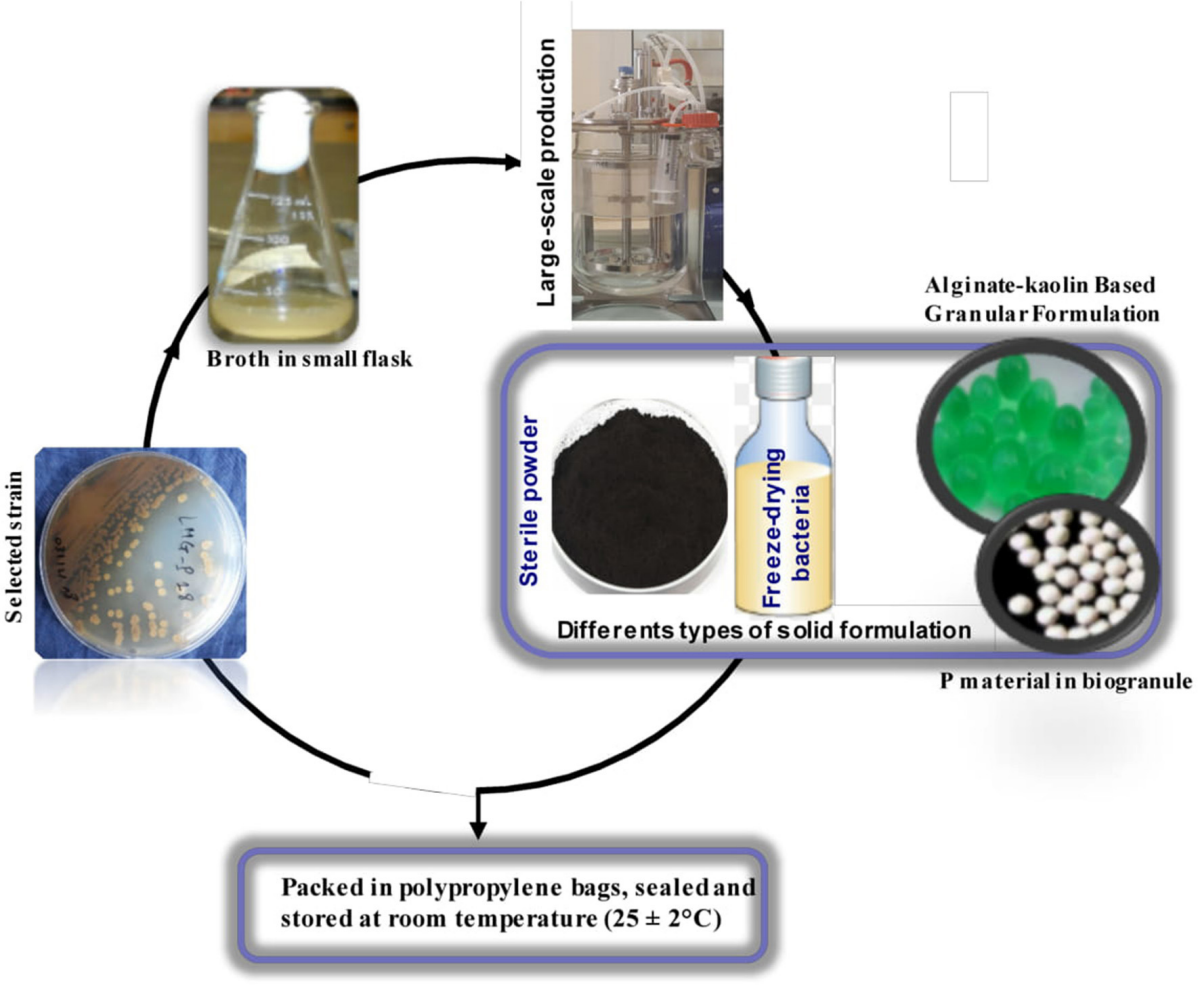
Simplified schematic flow chart for the production of *Actinobacteria* biofertilizer. Quality control is done at all stages of production a production as previously described (II.3).

These bacteria are characterized with, thermo-tolerant, drought-tolerant, easier to cultivate and formulate, easier to store and transport, and extended shelf life. *Actinobacteria* spores can be stored for a long time because they are very resistant to several environmental factors (Sharma et al., 2014). Dry formulations, such as powders containing mostly spores or granules can be manufactured with simple and cost-effective time technology. On the other hand, while most P-solubilizing organisms act through a mechanism of acidification of the soil, *Actinobacteria* appears to be moreover disposed of by other, efficient mechanisms such as chelation (Delvasto et al., 2006; Sharma et al., 2013; Nandimath et al., 2017). The high buffering capacity of the soil, generally prevents acidification and causing a failure of some biofertilizers (Khan et al., 2007). Irrespective of the type of soil, it acid or alkaline, *Actinobacteria* are able to solubilize rock phosphate (RP) and potassium rock (Hamdali et al., 2008). Therefore, a biofertilizer based on *Actinobacteria* inoculated into these materials will provide continuous supply of P and K for sustainable plant growth. In addition to their fertilizing ability, *Actinobacteria* can be used as biopesticide and bio-immunizing agents against plant diseases (Majeed et al., 2015; Barka et al., 2016). In fact, some *Actinobacteria* limit Fe availability in the soil by producing a Fe-chelating agent, siderophore. This results in low availability of this element, which limits or suppresses the growth of pathogens and their ability to cause diseases (Solanki et al., 2012; Raimi et al., 2017). Otherwise, the sequestration of these cations by the excreted siderophores led to the solubilization of the insoluble phosphates. The figure below summarizes in a very simple way the different stage of the bioformulation of *Actinobacteria* as a fertilizer (Figure 3). Some *Actinobacteria*, in addition to their biofertilization effects (ability to solubilize RP) can act as growth-promoting rhizobacteria (PGPR) as like other PGPR through phytohormones production such as indole acetic acid (IAA), gibberellins and cytokinins (Gopalakrishnan et al., 2014; Sousa and Olivares, 2016). A large amount of IAA (222.75 ppm) is produced by *Actinobacteria* belonging to the genus of *Nocardiopsis* and isolated under the rhizosphere of mandarin (Shutsrirung et al., 2013). IAA is one of the main phytohormone enhancing plant growth by stimulating the elongation of stems and roots. Other *Actinobacteria* belonging to the genus of *Streptomyces* have also shown a positive effect on the growth and elongation of common bean roots (Igarashi et al., 2002). Similar results were reported by Rashad et al. (2015) who showed that marine *Actinomyces* produce lot of phytohormones, including gibberellic acid. These phytohormones have been tested on eggplant (*Solanum melongena*) and have improved the agronomic performance of the species. On the other hand, Endophytic *Actinobacteria* produces secondary metabolites that improve growth and resistance to various environmental stresses (Girão et al., 2019). For instance, kasugamycin and mildiomycin from *Streptomyces kasugaensis* and *S. rimofaciens* respectively protect against rice blast and powdery mildew diseases (Sathya et al., 2017). According to Srivastava et al. (2015), PGP-*Streptomyces rochei* SM3 increase chickpea biomass accumulation (20%) and induces stress tolerance against NaCl and *Sclerotinia sclerotiorum*. Previously (Hamdali et al., 2008) reported that *Micromonospora aurantiaca*- *and Streptomyces griseus* increase wheat shoots and roots weight, respectively 50–47% and 80–78. Using two P-solubilizing actinomycetes as biofertilizers, Mba (1994) showed that these strains led to a 43% increase in soybean yield and soil properties were also improved. Alam et al. (2012) achieved an increase in yield (11%) of geranium herbs inoculated with *Streptomyces sp*. The ability of some *Streptomyces* to fix nitrogen as free-Living is an additional advantage in offsetting nitrogen losses from soil (Dahal et al., 2017). It has been documented that *Actinobacteria* promotes legume symbioses through hyphal elongation for symbiotic fungi (Schrey and Tarkka, 2008) and /or by increasing nodulation for nitrogen fixing symbiosis (Sathya et al., 2017). The multifunctionality of *Actinobacteria* is a definite advantage in the prospect of using them as bioinoculants for sustainable agriculture. However, beneficial PGP traits of *Actinobacteria* require more extensive research and more field trials. Also, extensive studies on *Actinobacteria* mechanisms of PGP action are needed.

**Figure 4:**
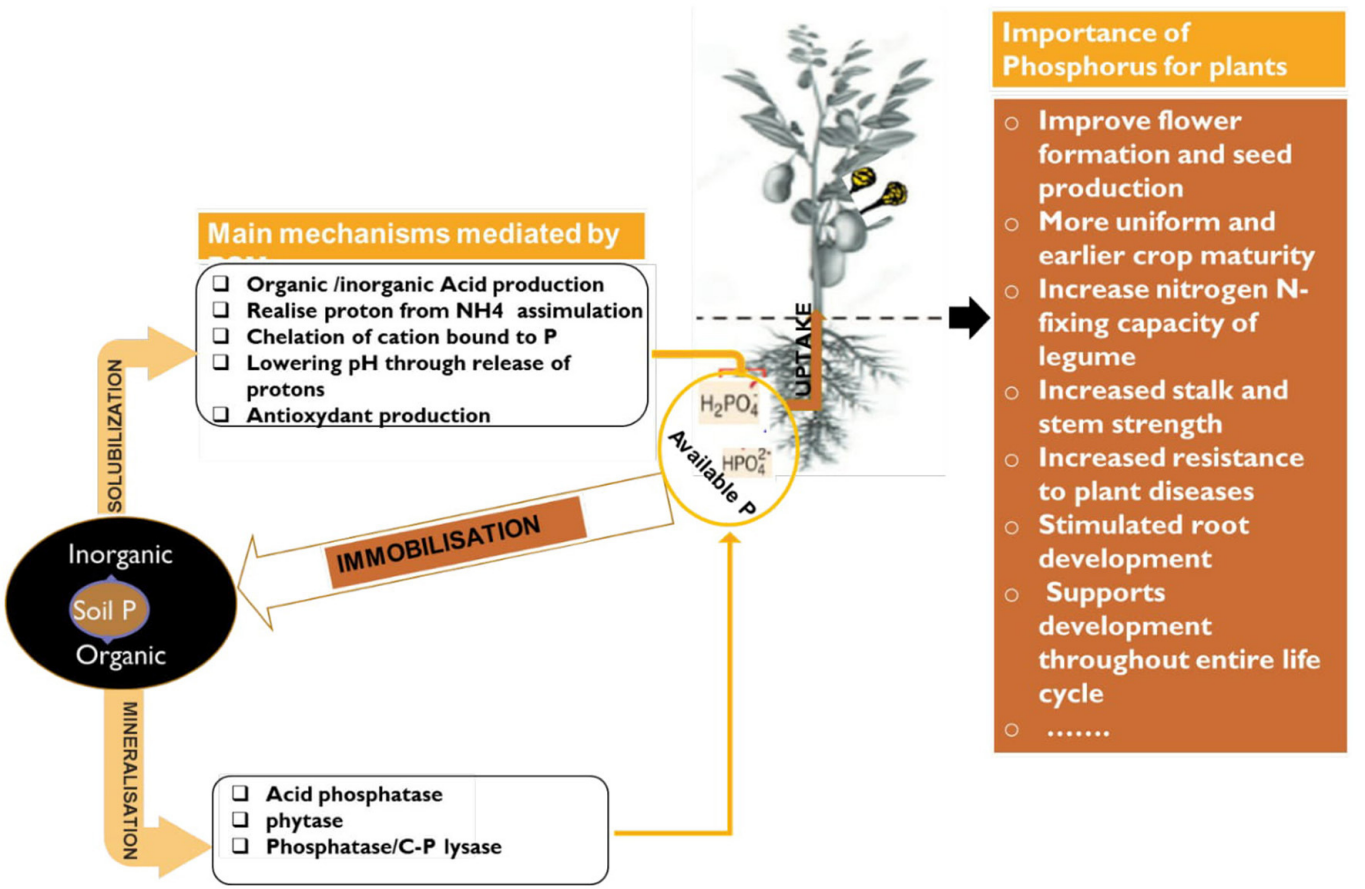
Schematic representation of P solubilization/mineralization/immobilization by PSM and its importance for plant growth.

**Figure 5:**
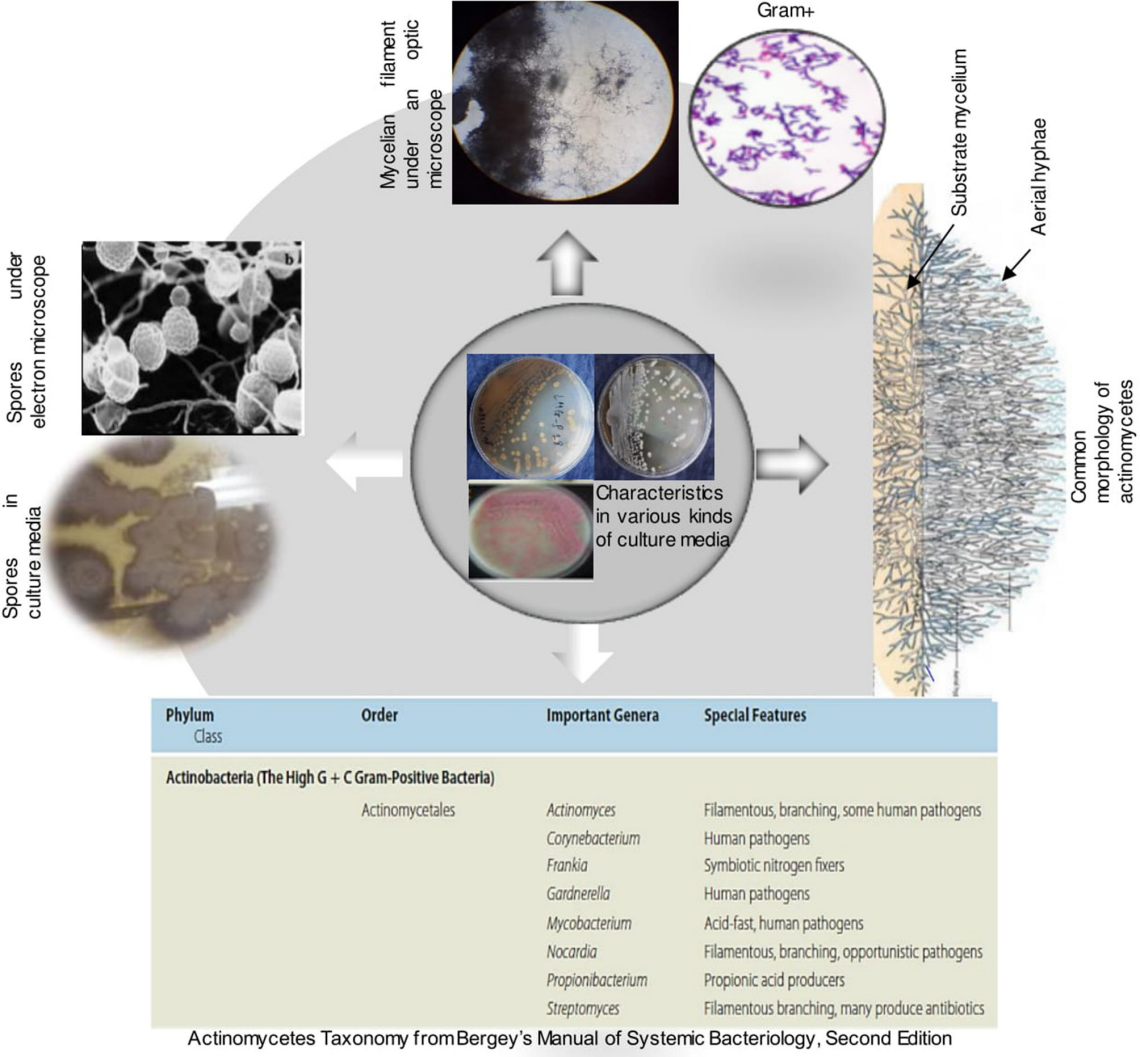
Some features of actinobacteria (Bergey's Manual of systematic Bacteriology, Barka et al., 2016).

## Optimization of *Actinobacteria* For Mass Production

Bacterial production and formulation are close. Hence, it is important to find out how, when, where and under what conditions the selected bacteria for bioformulation work. For this, prior optimization of the bacteria by monitoring growth parameters such as pH, culturing conditions, temperature, incubation time etc. is required. Many *Actinobacteria* species are grown on the common bacteriological media under normal conditions i.e., pH 7, T°C 28–30°C (Pudi et al., 2016). However, some species belonging to *Frankia* genus require special incubation conditions and growth media. The maximum yield is usually obtained after 7–10 days of incubation. For sporulating *Actinobacteria*, modification of nutrient parameters can induce and increase spore yield. In order to reduce the use of chemicals for mass production, several cost-effective substrates have been tested successfully. For instance, Soares et al. (2007) and Diraviyam et al. (2011), have shown that sterile moistened rice, rice bran, wheat bran and sugarcane residues can be viable methods for inoculum mass production of *Actinobacteria*. Despite of this innovation, research must be continued to test natural, inexpensive and available products for microbial biomass production. After mastering mass production, validation of the inoculation methods is also an essential step for biofertilizer. Three main biofertilizers inoculation methods (seeds treatment, root dipping and soil application) are generally used. For *Actinomyces*, better plant growth and yield are found when inoculum has been applied to the soil or substrate before sowing seeds (Gopalakrishnan et al., 2012; Sousa and Olivares, 2016). According to these authors, this type of application leads to a better establishment of the inoculated strain and better colonization of soil and/or roots. However, more field research results are still needed to optimize inoculation techniques in actinobacterial biofertilizers.

## Environmental Risk/Safety of *Actinobacteria*

There are few publications on the biosafety of biofertilizers, especially those based on *Actinobacteria*. Most of *Actinobacteria* fall in biosafety level 1, i.e., they are not known to be responsible for diseases (Shepherd et al., 2010). Only, few microorganisms used as bio fertilizers fall in biosafety level 2, i.e., opportunistic pathogens and presents health and/or environmental risk. This does not exclude the respect of good microbiological practices by following general laboratory safety rules. In this regard, research and industrial laboratories must verify the level of pathogenicity or any other risk related to microorganisms before embarking on a process of bioformulation or commercialization.

The environmental risks of inoculation are related to the fact that exotic microorganisms can disrupt the functioning of the soil microbiome because they can create invasive and competitive reactions (Glick, 2012; Sathya et al., 2017). Given these risks, it is more recommended to use native isolates because they have lower ecological risk to ecosystem. Otherwise, prior field studies must be carried out. On the other hand, *Actinobacteria* are major producers of antibiotics, so the horizontal transfer of genes between bacteria might be particularly risky in case of resistance (Egan et al., 2001). In a study, Martinez (2008) had already warned about the risk of disruption of rhizospheric population and resistance selection due to a large release of antimicrobial compounds. Additionally, about ten species belonging to genus of *Streptomyces* are known to be plant-pathogens and responsible of the common scab of potatoes (Labeda, 2011; Vurukonda et al., 2018). Such species should never be subject to bioformulation for biosecurity issues.

## Conclusions and Perspectives

One of the main problems to be solved in the next decades is to minimize dependence on phosphate fertilizers. To meet this challenge, PSMs must play a bigger role. It is known and well-documented that PSMs are able to solubilize rock phosphate. Exploitation of this potential may be a promising technique for plant phosphate nutrition. Thus, several tests of selection, identification, performance, and bio-formulation have been carried out in recent past to meet this need. However, this review shown that most of the experiments are carried out at laboratory or greenhouse scales and few papers have presented results in the field. So, we suggest some future lines: (i) since we know that results in laboratory or greenhouse conditions do not always reflect those of the field, therefore, consistent field results are a prerequisite for a massive adoption of biofertilizers based PSMs; (ii) there is also need for further research to understand the complex associations between the microbes-rock phosphate, dynamics of microbial populations, mechanisms of PSMs, and suitable technique of inoculation according the target crop and/or stain.

These data will allow to model the process of solubilization and even bioformulation (Saeid, 2018); (iii) the market of biofertilizers based on PSMs is very low compared to the biofertilizer based on nitrogen-fixing bacteria, 14 vs. 79% according to Transparency Market Research (2014). Therefore, potential problems associated with quality and stability of PSMs inoculum should be investigated with new approaches stemming from the collaboration between microbiologists, materials scientists and agricultural scientists (Arora, 2015; Berninger et al., 2018). Additionally, inoculation techniques and methodology should be described in detail to allow repetition of the experiment (Bashan et al., 2016b); (iv) among PSMs, *Actinobacteria* are the least studied for their agronomic interest. These bacteria deserve special attention due to their ability to adapt to diverse ecosystems such as desert soil, marine soil, rock phosphate deposit etc. (Nafis et al., 2019; Qu et al., 2019). Therefore, future research should focused on this group which still has a hidden repertoire that can be explored to develop quality bio-fertilizers. By associating these bacteria with Arbuscular mycorrhiza fungi, they can solubilize and mobilize P stored within the soil resulting in efficient biofertilizer. It is also necessary to focus on multi-functionality of *Actinobacteria* including nitrogen fixers and solubilizing phosphorus since plant production is largely dependent on N and P availability. *Actinobacteria* biotechnology has the potential to induce profound modification of agricultural practices, if it is developed. Therefore, there is a need for disruptive innovation and scientific research to ensure optimal rock phosphate solubilizing and bioformulation for sustainable and ecofriendly agriculture. An optimization of the processes of selection, multiplication, storage of PSM and their interactions in the rhizosphere are steps to master in order to develop efficient microbial inoculants with high phosphorus solubilization capacity.

## Author Contributions

All authors listed have made a substantial, direct and intellectual contribution to the work, and approved it for publication.

### Conflict of Interest

The authors declare that the research was conducted in the absence of any commercial or financial relationships that could be construed as a potential conflict of interest.
